# Supplementary material for: Conditional chemoconnectomics (cCCTomics) as a strategy for efficient and conditional targeting of chemical transmission
Source: eLife. 2024 Apr 30;12:RP91927. doi: 10.7554/eLife.91927 (PMC11060718; doi:10.7554/eLife.91927)
Supplement: Figure 3—source data 3. [file elife-91927-fig3-data3.zip › Figure 3 source data3 part 1.zip!/Figure 3-Source data 3-part1/1175-9-CRISPResso_Report_rtnd62ko.zip!/CRISPResso_on_1175_11/3b.Insertion_deletion_substitutions_size_hist.pdf]

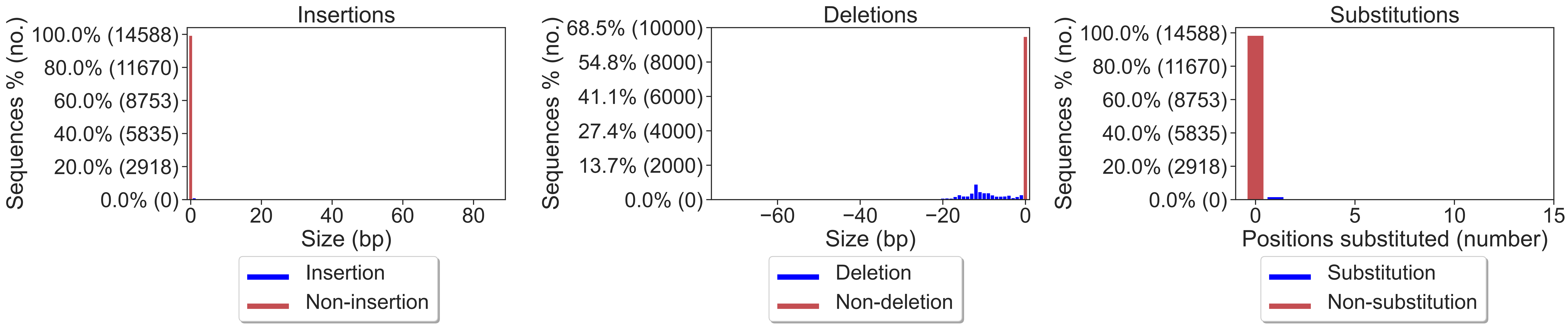
Insertions
Sequences % (no.)
100.0% (14588)
80.0% (11670)
60.0% (8753)
40.0% (5835)
20.0% (2918)
0.0% (0)
0
20
40
60
80
Size (bp)
Insertion
Non-insertion
Deletions
Sequences % (no.)
68.5% (10000)
54.8% (8000)
41.1% (6000)
27.4% (4000)
13.7% (2000)
0.0% (0)
−60
−40
−20
0
Size (bp)
Deletion
Non-deletion
Substitutions
Sequences % (no.)
100.0% (14588)
80.0% (11670)
60.0% (8753)
40.0% (5835)
20.0% (2918)
0.0% (0)
0
5
10
15
Positions substituted (number)
Substitution
Non-substitution
